# Rural physician deployment programs in five Southeast Asian countries: a policy and narrative review

**DOI:** 10.1186/s12913-026-14708-7

**Published:** 2026-05-22

**Authors:** Thi Thuy Dung Nguyen, Thi Nguyet Linh Pham, Quang Minh Ngo, Anh Duc Nguyen, Xuan Hung Pham, Le Minh Anh Tran, Duy Nguyen, Nguyen Xuan Quynh Truong, Duc Huy Le, Thanh Tung Pham

**Affiliations:** 1 College of Health Sciences, VinUniversity, Hanoi, Vietnam; 2 Center for Innovations in Health Sciences, VinUniversity, Hanoi, Vietnam; 3 Research Advancement Consortium in Health, Hanoi, Vietnam; 4 Department of Epidemiology, Harvard TH Chan School of Public Health, MA, USA; 5 Health Alliance for Diversity, Equity and Inclusion – HADEUS, Hanoi, Vietnam

**Keywords:** Rural health services, Rural physician deployment, Health workforce, Southeast Asia, Rural health policy

## Abstract

**Introduction:**

Access to healthcare – particularly the availability of physicians – remains a persistent challenge for rural populations due to longstanding urban–rural workforce imbalances. This issue is especially pronounced in Southeast Asia’s low- and middle-income countries, where rural communities make up a substantial share of the population. In response, several governments have implemented rural physician deployment programs; however, limited documentation and evaluation of these initiatives hinder meaningful cross-country comparison. This review provides a comparative synthesis of the design, incentives, retention patterns, and impacts of such programs in Thailand, Malaysia, Indonesia, the Philippines, and Vietnam.

**Method:**

A policy and narrative review was conducted, incorporating systematic search procedures guided by the PICO framework. Relevant literature was identified through PubMed, Google Scholar, and grey literature sources (including government documents such as government decrees, administrative reports, and program evaluations papers) focusing on physician deployment programs in countries with comparable socioeconomic characteristics.

**Results:**

Findings revealed substantial variation in program design. Thailand and Malaysia implemented mandatory service schemes, whereas Indonesia, the Philippines, and Vietnam adopted voluntary or semi-compulsory approaches. All five countries utilized financial and career advancement incentives to attract and retain physicians in rural posts. Thailand achieved the highest reported physician retention rates – 52.5% in the conventional track and 78.2% in targeted recruitment programs – while the Philippines reported 18%. Retention data for Indonesia, Malaysia, and Vietnam were limited or unavailable. Improvements in healthcare coverage and access were noted in Thailand and Indonesia, while the Philippines’ program demonstrated measurable improvements in maternal and child health outcomes. Vietnam also reported increased physician presence in underserved areas, although comprehensive impact data were lacking. Malaysia’s program structure was well-defined but lacked evaluative evidence.

**Conclusion:**

Rural physician deployment programs in Southeast Asia share common structural elements but differ substantially in service requirements, incentives, and implementation strength. Thailand’s integrated recruitment and incentive model shows the clearest evidence of effectiveness, while other countries face gaps in retention data and evaluation. Strengthening routine monitoring and systematic impact assessment is needed to guide future improvements in rural workforce policy across the region.

**Supplementary Information:**

The online version contains supplementary material available at 10.1186/s12913-026-14708-7.

## Background

Despite global progress towards Universal Health Coverage, access to healthcare remains a significant challenge for rural population across countries in Southeast Asia [1], especially the low- and middle-income countries (LMICs). According to the 2024 Association of Southeast Asian Nations (ASEAN) Statistical Brief, only four countries (Singapore, Brunei, Malaysia, and Indonesia) met the WHO’s 2016 benchmark of 44.5 health workers per 10,000 population, while Vietnam and Thailand reached only the earlier 2006’s threshold of 22.8 [2, 3]. The fact that most lower-income ASEAN countries still remain far below these levels highlights a deep regional inequity. This disparity is further compounded by urban–rural divides: across the 11 Southeast Asian countries, an estimated 46.3% of the population lived in rural areas in 2023 [4], where access to healthcare services is particularly limited. This imbalance is driven by a combination of structural and systemic barriers to achieving health equity for rural population, including workforce concentration in urban centers [5], lack of resources and infrastructure [6], migration of physicians to higher-income settings [1], and insufficient research into rural health policies [5, 7]. These issues are further compounded by policy implementation gaps, notably the weak monitoring and evaluation of national rural deployment initiatives. Despite the implementation of the “2010 WHO global policy recommendations to improve rural health worker recruitment and retention” [8] and the WHO South-East Asia Region’s commitment to the objectives of “Decade for health workforce strengthening (2015–2024)” [9], most countries implemented several of the recommended interventions but without establishing baseline data or mechanisms to monitor progress [5]. Consequently, evaluations of implementation outcomes were limited, and the absence of systematic monitoring hindered timely policy adjustments.

The key challenge, therefore, lies in the absence of comparative analysis across countries to inform policy design and refinement. While several Southeast Asian governments have introduced rural physician deployment programs, their approaches differ substantially. Yet, little is known about the relative effectiveness of these strategies, and few reviews have systematically synthesized the evidence on their structure, implementation, and outcomes. This review addresses this gap by systematically comparing the structure, incentives, physician retention, and health outcomes of rural deployment programs in five Southeast Asian lower-middle and upper-middle-income countries: Thailand, Malaysia, Indonesia, the Philippines, and Vietnam.

Our guiding research question is: What are the structural characteristics, incentive strategies, and outcomes of rural physician deployment programs in selected Southeast Asian countries; and from that, what lessons can inform workforce policy and education in similar socioeconomic contexts? By offering a structured synthesis of diverse program models, this review aims to inform health workforce planners, educators, and policymakers seeking to design or improve context-appropriate rural deployment strategies.

## Methods

A policy and narrative review were conducted using systematic procedures to examine the structure, incentives, retention, and health system impacts of rural physician deployment programs in five Southeast Asian countries: Thailand, Malaysia, Indonesia, the Philippines, and Vietnam. The selection and organization of analytical domains were guided by the WHO 2010 Global Policy Recommendations on Increasing Access to Health Workers in Remote and Rural Areas through Improved Retention [8]. While other broader health labour market and person-centred frameworks provide valuable insights into structural and contextual determinants of rural workforce retention, the WHO 2010 framework was selected for this review because it offers a systemic, structured, and evaluative logic linking intervention design to recruitment, retention, and system-level outcomes, which is aligned with the comparative focus of this study. It also represents the most widely recognized global normative model specifically developed to guide rural health workforce retention policies, particularly in low- and middle-income country contexts, and provides a framework suitable for cross-country policy comparison. The framework categorizes rural retention strategies into educational, regulatory, financial, and professional support interventions, and proposes a systems-based evaluation model linking policy design to outputs, outcomes, and long-term impact. In this review, we operationalized these domains into four comparative categories: program structure, incentive mechanisms, retention outcomes, and impacts. Educational and regulatory interventions (e.g., compulsory service, bonded scholarships, targeted rural recruitment) were synthesized under program structure, while financial and professional support measures were grouped as incentives. Consistent with the WHO inputs-outputs-outcomes-impact framework, we distinguished between workforce-level outcomes (e.g., recruitment and retention) and two levels of impact: (1) improvements in rural health workforce availability and service coverage, corresponding to system performance outcomes, and (2) changes in rural health indicators, reflecting longer-term population health impact. These domains are theoretically grounded in the WHO 2010 framework, which helps conceptualize rural retention interventions as operating through distinct governance levers (education, regulation, financial and professional support) and links them to sequential levels of effect, from recruitment and retention outputs to workforce performance and population health impact. This systems-based logic allows the intervention to be examined along the causal chain from policy design to health outcomes.

The five countries were included as they had implemented structured rural physician deployment or had available evaluative evidence. Other countries in the region, such as Laos, Cambodia, and Myanmar, remain at the policy-proposal stage [10–12], while Brunei, and Timor-Leste lack documentation of any deployment programs; Singapore was excluded as a high-income country.

**Fig. 1:**
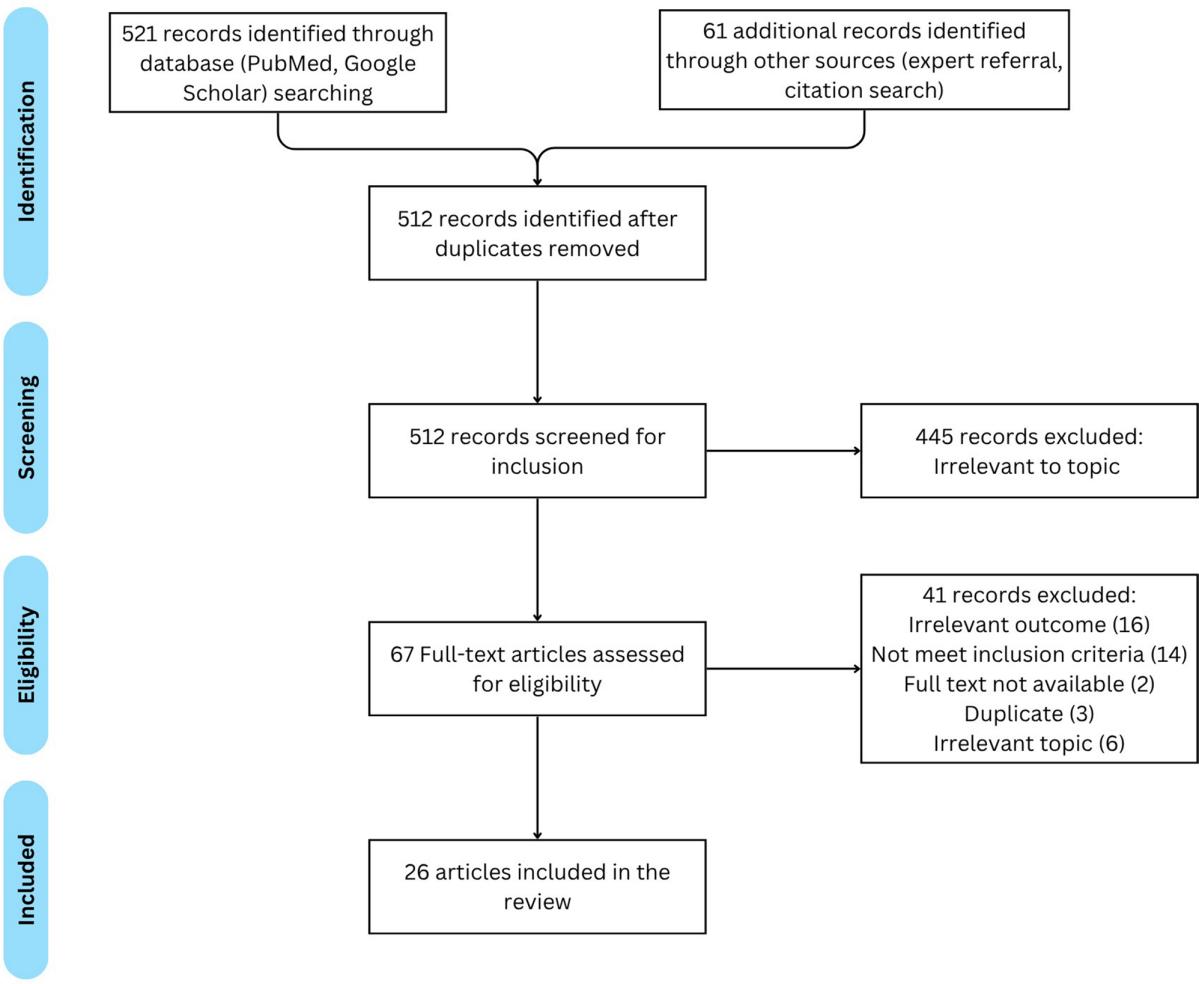
PRISMA flow diagram

A narrative review methodology was chosen due to the diverse study designs and the need for interpretive analysis across diverse health system contexts. This approach also allows for flexibility in synthesizing policy-oriented evidence while maintaining rigor in the search and selection process [13]. Although this review followed a structured search and transparent screening process (Fig. 1) to enhance reproducibility; however, its objective was not exhaustive evidence mapping or application of formal risk-of-bias tools. Therefore, it is best classified as a narrative policy review, synthesizing descriptive and contextual information on rural health workforce retention policies.

Following methodological guidance for narrative reviews that adopt systematic principles, we developed a comprehensive search strategy based on the PICO framework (Participants, Interventions, Comparators, Outcomes) to ensure that the key elements of the question are well defined [14]. Search keywords included physician, rural health services, mandatory rural program, retention, impact, and specific country and program names. The literature was searched using PubMed, Google Scholar, and grey literature sources (including government documents such as ministry decrees, administrative reports, and program evaluation papers). The included publications time ranged from 1999 to 2024, with most sources published between 2010 and 2024, reflecting the period during which rural physician deployment programs were formally implemented and evaluated across the selected countries. Literature searches were conducted using structured keyword combinations that paired each country name with policy- and workforce-related terms. Core search patterns included combinations such as (“[country]” AND “rural physician deployment”), (“[country]” AND “mandatory rural service” OR “rural doctor program”), and (“[country]” AND (“Ministry of Health” OR “rural health policy” OR “human resources for health” OR “rural health services” OR “health system”)). These structured combinations were applied across PubMed and Google Scholar, with country names sequentially replaced by Thailand, Malaysia, Indonesia, the Philippines, and Vietnam. Grey literature was identified through: Official Ministry of Health websites (Thailand, Malaysia, Indonesia, Philippines, Vietnam), national legal decree repositories, WHO and World Bank country reports, citation chaining from included studies, and expert referral for policy documents not indexed in databases. These sources were reviewed for information on physician workforce deployment and rural service policy contexts. The search strategy and terms are presented in Supplementary Table 1.

We included studies and reports that (1) described national or subnational rural physician deployment programs, and (2) provided accessible full texts. We included policy documents, legal decrees, administrative reports, narrative program descriptions, and evaluation studies (qualitative or quantitative) that provided substantive information on program structure, incentives, implementation, or outcomes. Primary searches were conducted in English. Where key policy documents were not available in indexed English-language sources, relevant non-English government documents were identified through targeted grey literature review and professional networks, translated to English, and included if they provided substantive program information. We excluded sources that were not publicly available in full-text format and those focused on countries outside Southeast Asia, with high-income status, or with undocumented programs. Five independent reviewers divided the articles among themselves and screened titles, abstracts, and full texts for inclusion. Discrepancies were resolved through discussion with a sixth reviewer. The study selection process is illustrated in the PRISMA flow diagram (Fig. 1). Data extraction was conducted using a structured template capturing program design, incentive mechanisms, physician retention rates, and documented health outcomes. Although formal risk-of-bias tools were not applied, included sources were reviewed for clarity, relevance, and adequacy of policy description to ensure they provided sufficient information for comparative analysis. For official documents and grey literature (government decrees, ministerial reports, WHO or World Bank reports), credibility was established through their institutional origin rather than formal appraisal tools. Findings were synthesized thematically, organized around the key dimensions of deployment program structure, incentives, retention, and impact. Patterns, gaps, and country-specific variations were identified and discussed to inform implications for rural health policy in the region.

## Results

### Study selection and overview

The systematic search yielded a total of 26 sources meeting the inclusion criteria, comprising peer-reviewed journal articles (*N* = 13) and grey or official documents (*N* = 12), including government or intergovernmental documents and reports, program evaluations, and legal decrees, and one academic book (*N* = 1). These sources documented physician deployment programs across the five target countries: Thailand, Malaysia, Indonesia, the Philippines, and Vietnam. The selection process is illustrated in the PRISMA flowchart (Fig. 1), and the full list of included sources with extracted data is presented in Supplementary Table 2. A summary of programs’ structure, incentives, recruitment and retention, and impacts are presented in Supplementary Table 3, enabling clearer comparison of policy design and evolution over time.

### Program structure

Across the five countries reviewed, physician deployment to rural areas was implemented through either compulsory or voluntary/semi-compulsory service schemes.

In Thailand and Malaysia, rural service is mandated as part of the professional pathway following medical school graduation [15, 16]. Thailand introduced a nationwide compulsory rural service policy in 1972, requiring all public medical graduates to serve in rural areas for three years [17]. To strengthen long-term rural workforce capacity, Thailand also developed targeted recruitment tracks (special tracks) such as the Collaborative Project to Increase Production of Rural Doctors (CPIRD) and One District One Doctor (ODOD). CPIRD focuses on admitting provincial secondary students for medical training tied to a three-year rural service obligation, while ODOD targeted more remote areas and extends this commitment to up to 12 years and offers full scholarships, guaranteed placement in the student’s home district, and other retention incentives [18, 19]. Similarly, Malaysia implements a compulsory public service, requiring doctors to complete a minimum of two years of public service as medical officers as proposed in the Medical Act 1971 [20–22]. This service takes place after completing a five-year undergraduate medical program and a minimum of 2–3 years of supervised housemanship training as house officers, upon which they receive full registration by the Malaysian Medical Council as medical officers [20, 21, 23]. Although Malaysia’s Medical Act (1971) does not explicitly stipulate compulsory rural public service, the ePlacement 2.0 portal – launched by the government for registration to medical officer postings – requires medical officers to indicate placement areas in Sabah or Sarawak, two predominantly rural and underserved states [24]. This administrative allocation effectively makes rural postings the default option for newly appointed contract medical officers.

By contrast, Indonesia, the Philippines, and Vietnam operate physician deployment programs with voluntary or semi-compulsory participation. Indonesia’s Pegawai Tidak Tetap (PTT) program was initially compulsory for three years (since 1992), but transitioned to a six-month optional program (Nusantara Sehat/ NS program) in 2007 following resistance from graduates [25]. The Doctors-to-the-Barrios (DTTB) program in the Philippines offers a two-year rural placement to incentivize service in underserved municipalities [26]. In Vietnam, Decision No. 585/QĐ-BYT (2013) stipulates minimal rural deployment periods of three years for male, and two years for female, but participation remains partially voluntary and focused on specific priority regions [27]. In 2020, an amendment to this policy specified that young doctors participating in the 585 Program who had already been recruited by local health facilities in these areas must serve at least five years at their recruited institutions after completing Specialist Level I training [28].

### Incentives

Incentive structures across the five countries varied considerably in form and amount, but commonly and primarily targeted two domains: financial compensation and career advancement opportunities (see Supplementary Table 3). Values are presented as reported in source documents. Due to differences in implementation years and currency units, figures are not inflation- or exchange-rate-adjusted; comparisons are therefore approximate.

#### Career promotion and scholarship opportunities

Rural services were considered a prerequisite for residency in Thailand [29]. Completion of rural services enabled physicians to apply for a Royal Thai Government scholarship to cover for the total specialty training expense (approximately 12,000 USD) [15]. Additionally, a PhD-equivalent degree for specialty in preventive medicine, general practice and family medicine could also be obtained after five years of rural service [15], which was awarded as a Certificate of Proficiency (called the Thai Board certificate) from the Medical Council of Thailand [29]. Furthermore, special recruitment track ODOD offered full scholarship covering tuition fee and medical expenses during their medical training [19].

With regards to Malaysia, before 2015, completing compulsory service guaranteed an automatic career promotion to UD-48 (mid-grade career – equivalent to 5 years of postgraduate experience) and access to federal scholarships for clinical specialist training [30, 31]. However, the rapid expansion of medical schools and prolonged housemanship training led to an oversupply of graduates, making it unfeasible for the government to offer permanent posts to all who completed housemanship [21, 32]. Beginning in December 2016, under what became known as the Contract Doctor System, most Medical Officers (MOs) were appointed on a contractual basis rather than permanent posts [22, 32, 33]. This approach aimed to shorten the waiting period (up to eight months) for housemanship and service placement by allowing the ministry to allocate contract posts flexibly based on facility capacity [21, 22]. Despite that, under this policy, most MOs were not automatically eligible for automatic promotion or specialist scholarships [20, 34].

In Indonesia, upon completion of the rural services, physicians receive priority in subsequent civil recruitment process and are eligible for specialist training scholarships [25, 35].

After completion of DTTB program in Philippines, physicians could practice as epidemiologists, pursue public health research, or underwent residency training (public or private hospital), or remained in assigned municipality [26]. Additional incentives included Scholarship for Master’s degree in Public Management [26, 36].

In Vietnam, physicians deployed under Decision No. 585/QĐ-BYT (2013) were offered direct hiring contracts with Ministry-affiliated hospitals, tuition coverage for specialist training and prioritized for medical licensing, and guaranteed reassignment after completing their service [27]. Moreover, they are prioritized for postgraduate education opportunities and considered for long-term retention, promotion, if they remained in the assigned locality [27].

#### Salary and financial compensation

In Thailand, physicians serving in rural areas were eligible for a base monthly top-up of approximately 400 USD, which could increase to 1,379 USD when combined with location-based allowances, but were subject to a 11,300 USD to 56,000 USD penalty for contract breaches [15, 19, 29].

Malaysia also offered financial assistance in terms of a 10–25% salary increase for medical officers serving in rural postings [30]. However, under the Contract Doctor System, the MOs remained at the lowest grade (UD41), earning about USD 1,700 (≈ RM 8,000) less annually and lacking key benefits such as unrecorded leave and housing or education loans compared with UD44 permanent peers [20]. In 2020, the government adjusted the pay scale for contract officers to UD43 (RM 5,000–5,200 ≈ 1,050 USD per month) after five years of service, yet it did not fully resolve pay disparities, as contract officers remained subject to a salary ceiling with no time-based increments, unlike their permanent peers [20].

In Indonesia, rural physicians received a base salary of approximately USD230/month (2,050,000 IDR, based on an exchange rate of ~ 1 USD = 9,000 IDR), with additional incentives ranging from 370 to 640 USD for placements in remote and very remote areas, respectively, bringing the total monthly salary to approximately 600 to 870 USD [37]. The incentives for specialist doctors are even higher, reaching 920 USD (8,300,000 IDR) if working in very remote areas, allowing them to earn approximately 1,150 USD per month [37].

The Philippines provided one of the region’s highest public-sector salaries for rural physicians at approximately 1,870 USD /month (as mentioned in an article in 2021) [26].

For Vietnam’s rural program, doctors received full salary, various allowances based on region and profession, and additional financial support.

#### Allowances and non-salary benefits

Other incentives of Thailand included remote hardship, non-private practice, non-official hour service, and long years of service [17].

Malaysia also offered allowances for duty, relocation, housing loans, and a parallel professional development program [30]. Yet, some of these benefits were cut short for MOs under the Contract Doctor System [20].

For Philippines, DTTB doctors received subsistence and laundry, housing and insurance, transportation, and other benefits as described in Magna Carta of Public Health Workers [26, 36].

In Vietnam, doctors received support such as travel reimbursements, housing and living conditions, on-call payments during the program duration, and potential Communist Party membership and land allocation if they retained in the area after completion [27].

### Recruitment and retention

Among the five countries reviewed, Thailand demonstrated the most robust physician retention outcomes. Under its dual-track deployment system, the conventional program reported a 52.5% retention rate, while the targeted recruitment track (special track) achieved a notably higher rate of 78.2% [19]. Physicians enrolled in the special track were found to be 2.4 times more likely to continue employment within the Ministry of Public Health compared to those in the conventional pathway [38]. In contrast, the Philippines reported a significantly lower retention rate of 18% following service under the DTTB program [39]. While the program succeeded in temporarily addressing staffing gaps in underserved areas, long-term retention within the public health system remained a persistent challenge.

Retention data for Malaysia, Indonesia, and Vietnam were either limited or not systematically reported. However, findings from the literature indicate that both Malaysia and Indonesia continue to experience difficulties in sustaining physician presence in rural areas following completion of initial service obligations [25, 30]. In Vietnam, the implementation of Decision No. 585/QĐ-BYT (2013) initially fell short of its 2025 target of deploying 500 doctors to disadvantaged areas [27]. The goal was later revised to 450 by 2025 and 600 by 2030 [28], with official reports confirming that the adjusted targets were ultimately achieved [40].

Further information on recruitment volumes and year-by-year deployment figures, where available and calculated by the reviewers, is provided in Supplementary Table 3.

### Rural health impacts

#### Improvement in coverage and access to healthcare

Rural physician deployment programs have contributed to improvements in healthcare coverage and provider availability in several Southeast Asian countries, although the scope and strength of the evidence vary.

Since the initiation of rural physician deployment programs, Thailand saw an increase in doctor-to-population ratios in rural areas. From 1965 to 2010, under the Medical Education for Students in Rural Area Project (MESRAP), which was later expanded into the CPIRD, the doctor-to-population ratio in rural areas increased from 1-to-7,000 to 1-to-5,750 [15]. Data on changes in the doctor-to-population ratio in rural areas of other countries remained limited, limiting a general comparison.

In Indonesia, improvements in provider coverage have also been observed. Between 2006 and 2010, the proportion of very remote health centers without a physician declined from 30% (of 8,000 centers) to 17% (of 9,000 centers), suggesting that the PTT & NS program contributed to expanding service availability in hard-to-reach areas [37].

In the Philippines, the DTTB program was associated with an estimated 33% increase in the likelihood that a municipality would have a physician assigned to its public health center [41].

In Vietnam, administrative reports from the Ministry of Health (MOH) documented the training and deployment of 699 doctors to rural and remote regions from 2013 to 2024 [40]. Of these, 444 physicians were placed in 89 disadvantaged or border districts across 22 provinces in the northern mountainous, central, and Central Highlands regions [40], contributing to an overall increase in rural health workforce presence.

By contrast, in Malaysia, there is currently no publicly available data or formal evaluation reporting the impact of the country’s rural physician deployment scheme on healthcare coverage or access.

#### Improvements in rural health indicators

Among the countries reviewed, the Philippines is the only country to provide direct evidence of health outcome improvements after the implementation of rural physician deployment program. A cost-effectiveness evaluation of the DTTB program, conducted jointly by the University of Michigan and the Philippines Department of Health, demonstrated significant positive impacts on child health: The program was associated with reduced mortality from diarrhea and pneumonia in children under five years of age, as well as a decreasing underweight in children [36]. Moreover, it doubles the odds of using modern contraceptives among poor women; and decreases the odds of being underweight by two-thirds among poor children [41].

In contrast, direct evidence linking rural physician deployment programs to measurable health outcomes remains limited for Thailand, Indonesia, Malaysia, and Vietnam. While these countries have documented improvements in physician coverage and accessibility, few studies have formally evaluated the impact of these programs on health indicators such as mortality, morbidity, nutritional status, or service utilization.

## Discussion

### Success factors

This review highlights that successful rural physician deployment in Southeast Asia relies on an interplay of structured policy design, targeted recruitment, and meaningful incentives. Thailand emerged as a leading example, demonstrating how a multi-faceted approach – combining compulsory service, rural-origin student recruitment (CPIRD, ODOD), scholarship support, and career progression pathways – can yield comparatively high physician retention rates in underserved areas [15, 29, 42]. Embedding rural service into medical education and residency eligibility further strengthened physician commitment to rural practice [15]. It is important to note that while Thailand reports higher retention rates compared to other countries in this review, differences in monitoring systems, data completeness, and outcome definitions limit direct comparability. Reported variations in effectiveness may therefore reflect differences in data availability as much as differences in program performance.

Malaysia’s compulsory model included structured incentives and assured career advancement; however, the absence of a clear link between rural service and promotion, coupled with frequent policy shifts and limited permanent posts, may have hindered assessment of its long-term effectiveness. In contrast, Indonesia and the Philippines relied on optional or semi-compulsory schemes with variable support structures, which likely contributed to lower retention rates. Vietnam’s program, while newer in implementation, showed promising reach but is yet to be systematically evaluated in terms of long-term impact or health outcomes. Overall, financial and non-financial incentives were central to program design across all countries, but Thailand stood out for its use of policy levers – such as training-linked scholarships and legal enforcement mechanisms – to ensure adherence and continuity.

Thailand’s success in rural physician deployment reflects design principles similar to those adopted in high-income countries such as Australia and Canada. Both contexts rely on a “rural pipeline” model, recruiting local students for rural-based training to increase return-to-service rates. For example, as Thailand adopt the CPIRD and ODOD, the Australian Government established the Rural Health Multidisciplinary Training (RHMT) program, which offers health students the opportunity to train in rural and remote communities via a network of training facilities such as rural clinical schools (RCS), and university departments of rural health [43]. As a result, more medical students spent a minimum of one RCS and graduates with the most rural clinical experience were also working more in regional, rural and remote areas [43]. Likewise, the Northern Ontario School of Medicine, Canada recruits students from Northern Ontario and similar backgrounds, offering distributed, community-engaged learning across 70 rural sites, with 61% of medical doctor (MD) graduates subsequently choosing predominantly rural family practice training [44]. These structural and educational similarities may help explain why Thailand, despite being an LMIC, has achieved rural retention outcomes comparable to other higher-income nations.

### Ongoing challenges

Despite documented successes in deployment and initial coverage gains, retention beyond the service period remains a significant challenge across the region. Even in Thailand, a substantial number of physicians leave rural posts after fulfilling their obligations, attracted by better remuneration and opportunities in urban areas or private sectors [15, 26]. Infrastructural constraints and limited access to continuous professional development, particularly noted in Malaysia, Indonesia, and Vietnam, deter sustained rural practice [16, 28, 30, 37]. The Philippines also reported challenges in retaining physicians due to logistical issues, security concerns, and inconsistent support from local governments [42] In Indonesia, physicians deployed to remote and rural areas face persistent challenges that extend beyond clinical practice to their personal and family lives - difficult living conditions, professional isolation, and limited support infrastructure undermine both the quality of care and physicians’ well-being [45, 46]. These enduring hardships have long been recognized as key barriers to recruiting and retaining doctors in rural regions.

Moreover, inadequate supervision and mentoring for young doctors in rural settings have raised concerns about quality of care. Newly graduated physicians, especially in remote or isolated areas, often lack the necessary guidance, potentially compromising service delivery [15, 26].

For Malaysia, available information on rural physician deployment remains limited and fragmented. The e-Placement 2.0 system, launched only in 2025, is the first official policy to explicitly stated that medical officers’ posts need to include at least one placement option in Sabah or Sarawak, which are predominantly rural regions [24]. Prior to this reform, no national regulation explicitly mandated rural postings, to our knowledge; rather, graduates were simply required to complete compulsory public service within the public sector. This highlights a lack of peer-reviewed policy literature explicitly defining the posting length and designated areas for medical officers, both under the former permanent scheme and the post-2016 contract system. Consequently, no measurable evidence of program outcomes or impact was identified. In addition, growing dissatisfaction among contract medical officers led to the formation of the Hartal Doktor Kontrak (HDK) movement on 23 June 2021, representing junior doctors’ strike to advocate for job security and equal career opportunities [20]. Most of these contract doctors, who had served as frontliners during the COVID-19 pandemic, faced uncertainty regarding permanent appointment and specialist training prospects [20]. This highlighted the urgent need for a long-term, transparent workforce strategy from the MOH to ensure stability, equitable career progression, and sustained service in underserved areas.

### Funding

The sustainability and effectiveness of rural physician deployment programs are closely tied to their funding mechanisms, which determine how long such initiatives can operate, scale, and retain health workers in underserved regions. Across the five Southeast Asian countries, most programs were predominantly financed through public budgets, though their fiscal efficiency and adaptability varied substantially.

In Thailand, government funding has consistently supported the CPIRD and ODOD tracks [18]. In Malaysia, the program is also funded through the MOH, with the 2026 Budget about to allocate RM 46.5 billion (≈ USD 9.8 billion) – its largest health budget to date – to expand 4,500 permanent positions for contract doctors [47]. In Indonesia, the physician deployment scheme is also financed by the MOH, supplemented by occasional district-funded placements or short-term contracts for visiting physicians [48]. However, limited fiscal autonomy and delays in central fund disbursement under programs such as Nusantara Sehat (NS) have constrained local implementation and program sustainability [48]. The program in the Philippines is also a nationally funded initiative under the Department of Health (DOH), which provides salaries and statutory benefits, while local government units (LGUs) supplement support through housing, honoraria, and logistical assistance [26, 41].

In Vietnam, the program was initially funded through the World Bank supported Health Professionals Education and Training for Health System Reforms Project (HPET) during Phase 1 (2013–2020). Since Phase 2 (from 2021), tuition and training costs have been co-funded by the private sector, specifically Vingroup via Thien Tam Foundation, while other incentives continue to be supported through national funding [49].

These variations highlight that while public financing remains the backbone of rural physician deployment in the region, hybrid or co-funded models – such as Vietnam’s public–private transition – may offer an alternative for flexibility and resilience. Sustained funding mechanisms, coupled with transparent governance and local co-ownership, are therefore essential to ensure continuity, cost-efficiency, and equitable coverage in rural health workforce programs.

### Limitations

The strength of this review lies in its structured synthesis of diverse program models using a systematic narrative approach grounded in the PICO framework. It offers a regional comparative lens that has been lacking in the literature and contributes to health workforce policy by identifying replicable elements of success.

However, several limitations warrant consideration. First, data availability varied considerably across countries. While Thailand and the Philippines provided relatively complete and evaluable data on physician distribution and health impact, evidence from Malaysia, Indonesia, and Vietnam was fragmented or descriptive. This disparity limited our ability to perform more granular comparisons across health indicators. Second, potential reporting and selection bias represent an important limitation of this review. The inclusion of heterogeneous sources, particularly grey literature and official policy documents, means that reported outcomes may reflect variable documentation standards and selective emphasis on program achievements. Differences in monitoring systems and non-standardized outcome definitions across countries further constrain comparability. Because this study is a narrative policy review, formal risk-of-bias assessment tools were not applied; instead, sources were reviewed for clarity and relevance. These methodological characteristics may temper interpretation of comparative effectiveness across schemes. Third, the exclusion of non-English and non-Vietnamese materials may have led to omission of locally relevant insights, especially from Malaysia and Indonesia. Finally, the financial incentive amounts reported across countries were derived from policy documents implemented in different years and denominated in various currencies. As inflation rates and exchange rates vary over time, direct cross-country comparison of the nominal monetary values should be interpreted cautiously. Standardizing all incentives to a single reference year would have required assumptions on historical consumer price index and exchange-rate trajectories, which may introduce further uncertainty. Therefore, we retained the original reported values and highlighted this limitation when interpreting financial magnitudes. These limitations reflect broader challenges in health systems research, including the lack of standardized evaluation metrics for workforce deployment programs. Nevertheless, the findings remain valuable in highlighting common implementation patterns, identifying gaps, and informing future data collection efforts. Future research should prioritize rigorous longitudinal studies and expand evaluations of program outcomes on health indicators and long-term retention in rural areas.

### Implication for policy, practice, and research

While most of the five countries reviewed have established structural components such as recruitment pipelines, incentive schemes, and service regulations, monitoring and evaluation mechanisms remain underdeveloped. Basic program information was generally available, but systematic evidence on physician retention and direct health impacts was scarce. These gaps underscore the need for governments to move beyond structural design toward measurable impact assessment. Future program planning should embed routine data collection and evaluation frameworks from the outset, enabling routine tracking of outcomes and cost-effectiveness. Longitudinal evaluations are essential to assess whether recruitment and financial interventions translate into sustainable rural retention. From a research perspective, cross-country learning collaboratives and standardized indicators could strengthen regional benchmarking, while evidence-informed feedback loops would support adaptive policy refinement. Ultimately, sustainable rural workforce development requires not only the integration of education, incentives, and career pathways, but also systematic evaluation to ensure that these investments yield measurable improvements in rural healthcare access and equity.

At a broader regional level, the findings of this review suggest that Southeast Asian countries could benefit from a more coordinated approach to rural physician retention, drawing on lessons from the WHO South-East Asia Region’s (WHO SEAR) Decade for Strengthening Human Resources for Health [9]. While the 2010 WHO recommendations provided a conceptual global framework for improving rural health worker recruitment and retention through educational, regulatory, financial, and professional or personal support interventions, the WHO SEAR Decade 2015–2024 translated this framework into a regional action agenda. Although the WHO South-East Asia Region is not identical to the group of Southeast Asian countries examined here, its agenda can serve as a useful implementation model for the Southeast Asian countries. To be specific, it lays an operational model to achieve universal healthcare goals through periodic national action plans, routine progress reporting (e.g. 2-year country report submission to WHO SEAR), stronger health workforce information systems development, and platforms for cross-country learning. National governments’ role is to develop and regularly revise context-specific action plans, implement bundled interventions, and report to the WHO regional office, while WHO provides normative guidance, technical assistance, regional peer-learning platforms, and support for strengthening health workforce informatics capacity for monitoring and evaluation [9]. In terms of evaluation and monitoring, progress could be tracked using a shared monitoring framework based on 14 standardized health workforce indicators, which was developed after the second regional progress review by WHO SEAR. These indicators cover domains such as health worker density and distribution, health professional education, health worker retention, HRH governance, and HRH information systems [50]. Consistent with the WHO 2010 framework, evaluation should extend beyond program inputs alone to assess the links between intervention design, implementation outputs, retention and distribution outcomes, and longer-term health and system impacts. Health impacts may be assessed using indicators aligned with the WHO health workforce framework for achieving health and well-being, such as maternal and newborn outcomes, communicable and noncommunicable disease outcomes, and access to essential medicines and vaccines [9]. Moreover, longitudinal impacts could be measured in proportionate increase in the percentage of GDP spent on HRH programs [9]. With regard to government actions and intervention designs, it is emphasized throughout the WHO SEAR progress reviews that bundled interventions should be key rather than isolated measures, as they are interlinked [9, 50–52]. At the same time, regional learning should not assume that a single model will work across settings: bundled incentives are likely to be more effective than isolated interventions, but their effects may differ across target groups (younger versus older health workers; doctors versus nurses) and health systems [51]. Thus, rural retention strategies should be applied fairly, informed by health workers’ perspectives, and situated within broader workforce planning and labour market dynamics. As suggested by WHO SEAR, health labour market analyses (HLMA) can be used as a tool to inform health, social, and economic policymaking by generating evidence on health workforce market dynamics [51].

## Conclusion

This review shows that rural physician deployment programs in five Southeast Asian countries share common design features but diverge markedly in implementation and outcomes. Thailand and Malaysia adopted mandatory rural service models, whereas Indonesia, the Philippines, and Vietnam implemented voluntary or semi-compulsory schemes with varied service durations and incentive packages. Across all five countries, financial and career-advancement incentives were central policy levers; however, Thailand’s comprehensive approach – combining substantial financial allowances, targeted rural-origin recruitment (CPIRD, ODOD), and the integration of rural service into residency eligibility – was uniquely effective, achieving the highest documented physician retention rates (52.5–78.2%), compared with 18% in the Philippines and limited or unavailable data for Malaysia, Indonesia, and Vietnam. Evidence of improved rural healthcare access and coverage was observed in Thailand, Indonesia, and Vietnam, while only the Philippines managed to provide direct evidence of gains in improved health outcomes, particularly maternal and child health. Yet, across the region, routine monitoring, rigorous impact evaluation, and transparent reporting remain underdeveloped, constraining policy learning and optimization. To move beyond short-term deployment toward sustainable rural workforce strengthening, countries should embed robust monitoring and evaluation frameworks, link incentives to clear career pathways, and systematically assess long-term physician retention and changes in rural health indicators. At a broader regional level, greater coordination, shared monitoring frameworks, and adaptation of implementation lessons may help Southeast Asian countries strengthen rural physician retention more systematically and equitably.

## Supplementary Information

Below is the link to the electronic supplementary material.

Supplementary Material 1

## Data Availability

All data generated or analysed during this study are included in this published article and its supplementary information files.

## Abbreviations

SEA: Southeast Asia
LMICs: Low- and middle-income countries
ASEAN: Association of Southeast Asian Nations
WHO: World Health Organization
CPIRD: Collaborative Project to Increase Production of Rural Doctors
ODOD: One District One Doctor
MESRAP: Medical Education for Students in Rural Area Project
MD: Medical Doctor
MOs: Medical Officers
PTT: Pegawai tidak tetap (Non-permanent Employee)
NS: Nusantara Sehat (Healthy Archipelago)
DTTB: Doctors-to-the-Barrios
MOH: Ministry of Health
HDK: Hartal Doktor Kontrak (Contract Doctors’ Strike)
RHMT: Rural Health Multidisciplinary Training
RCS: Rural Clinical School
HPET: Health Professionals Education and Training for Health System Reforms Project
SEAR: South-East Asia Region
HRH: Human Resources for Health
GDP: Gross Domestic Product
AIDS: Acquired Immune Deficiency Syndrome
HLMA: Health Labour Market Analysis
