# Supplementary material for: Rural physician deployment programs in five Southeast Asian countries: a policy and narrative review
Source: BMC Health Serv Res. 2026 May 22;26:1131. doi: 10.1186/s12913-026-14708-7 (PMC13480059; doi:10.1186/s12913-026-14708-7)
Supplement: Supplementary file 1 [file 12913_2026_14708_MOESM1_ESM.docx]

**SUPPLEMENTARY MATERIALS**

**Rural Physician Deployment Programs in Five Southeast Asian Countries: A Policy and Narrative Review**

*Thi Thuy Dung Nguyen, Thi Nguyet Linh Pham, Quang Minh Ngo, Anh Duc Nguyen, Xuan Hung Pham, Le Minh Anh Tran, Duy Nguyen, Nguyen Xuan Quynh Truong, Duc Huy Le, Thanh Tung Pham*

**TABLE OF CONTENTS**

[**Supplementary Table 1. Search Strategies and Terms used in the Review**	1](#_Toc206060077)

[**Supplementary Table 2. Included Studies and Extracted Data**	4](#_Toc206060078)

[**Supplementary Table 3. Result Summary**	14](#_Toc206060079)

[**References**	17](#_Toc206060080)

# **Supplementary Table 1. Search Strategies and Terms used in the Review**

| **Search String** | **Title** |
| --- | --- |
| (("Physicians"[Mesh]) AND ( "Rural Health"[Mesh] OR "Rural Health Services"[Mesh] )) AND "Thailand"[Mesh] | Mandatory rural service for health care workers in Thailand |
| "Thailand" AND ("Health System" OR "Rural Health Policy") | Retention of doctors in rural health services in Thailand: impact of a national collaborative approach |
| (("Physicians"[Mesh]) AND ( "Rural Health"[Mesh] OR "Rural Health Services"[Mesh] )) AND "Thailand"[Mesh] | Impact of a 20-year collaborative approach to increasing the production of rural doctors in Thailand |
| (("Physicians"[Mesh]) AND ( "Rural Health"[Mesh] OR "Rural Health Services"[Mesh] )) AND "Thailand"[Mesh] | Integrated strategies to tackle the inequitable distribution of doctors in Thailand: four decades of experience |
| (("Physicians"[Mesh]) AND ( "Rural Health"[Mesh] OR "Rural Health Services"[Mesh] )) AND "Thailand"[Mesh] | Equity of health workforce distribution in Thailand: an implication of concentration index |
| (("Physicians"[Mesh]) AND ( "Rural Health"[Mesh] OR "Rural Health Services"[Mesh] )) AND "Thailand"[Mesh] | Rural physician recruitment program in Thailand: factors associated with medical student enrollment and post-graduation intentions |
| ("health system" OR "human resources for health") AND "Malaysia" | Human Resources for Health Country Profile 2019-2021 Malaysia |
| ("house officers" OR "medical officers" OR "medical graduates") AND "Malaysia" | Contract doctors’ strike in Malaysia: A content analysis of the perception of medical fraternity and stakeholders on Facebook |
| ("health system" OR "human resources for health") AND "Malaysia" | Extended Executive Summary: Supply and Needs-Based Requirement Projections of Malaysian Human Resources for Health Using System Dynamics Approach (Doctor, Dentist, Pharmacist, Nurse, Assistant Medical Officer) 2016–2030 |
| (Identified via other sources): Additional grey literature obtained from expert referral | Permanent medical officers must choose a placement in Sabah or Sarawak |
| Mandatory housemanship program AND rural health AND Malaysia | Health Workforce Crisis: Recruitment and Retention of Skilled Heath Workers in the Public Health Sector in Malaysia |
| ("health system" OR "human resources for health") AND "Malaysia" | Malaysia Health System Review |
| ("house officers" OR "medical officers" OR "medical graduates") AND "Malaysia" | House officers’ specialist career choices and motivators for their choice– a sequential mixed-methods study from Malaysia |
| (Identified via other sources): Citation search from included article “House officers’ specialist career choices and motivators for their choice– a sequential mixed-methods study from Malaysia” | Alternate careers for medical graduates and house officers in Malaysia. |
| (Identified via other sources): Additional grey literature obtained from expert referral | Annual Report 2016, Ministry of Health, Malaysia |
| ("house officers" OR "medical officers" OR "medical graduates") AND "Malaysia" | Malaysian Medical Students’ Career Intention (MMSCI): a cross-sectional study |
| Physician AND mandatory rural service AND Indonesia | What Do Doctors Want? Developing Incentives for Doctors to Serve in Indonesia’s Rural and Remote Areas |
| Health Worker AND Recruitment AND Indonesia OR Results | Health worker recruitment and deployment in remote areas of Indonesia |
| Physician AND mandatory rural service AND Indonesia | New Insights into the Supply and Quality of Health Services in Indonesia |
| "Physicians"[Mesh] AND "Rural Health Services"[Mesh] AND "Philippines"[Mesh] | Factors affecting retention in the Philippine National Rural Physician Deployment Program from 2012 to 2019: a mixed methods study |
| "Physicians"[Mesh] AND "Rural Health Services"[Mesh] AND "Philippines"[Mesh] | Cost-effectiveness analysis of a physician deployment program to improve access to healthcare in rural and underserved areas in the Philippines |
| "Physicians"[Mesh] AND "Rural Health Services"[Mesh] AND "Philippines"[Mesh] | Assessment of factors influencing retention in the Philippine National Rural Physician Deployment Program |
| Rural physician program AND Philippines AND Impact AND Evaluation | Impact Evaluation of the Human Resource for Health Deployment Program (HRHDP) |
| (Identified via other sources): Additional grey literature obtained from expert referral | Decision No. 585/QĐ-BYT of 2013 approving the Project "Pilot Deployment of Young Volunteer Doctors to Work in Mountainous, Remote, Border, Island Areas, and Regions with Especially Difficult Socio-Economic Conditions (Priority Given to 62 Poor Districts)" issued by the Minister of Health. |
| (Identified via other sources): Additional grey literature obtained from expert referral | Decision No. 4359/QĐ-BYT of 2020 amending Decision No. 585/QĐ-BYT on the approval of the Project "Pilot Deployment of Young Volunteer Doctors to Work in Mountainous, Remote, Border, Island Areas, and Regions with Especially Difficult Socio-Economic Conditions (Priority Given to 62 Poor Districts)" issued by the Minister of Health. |
| (Identified via other sources): Additional grey literature obtained from expert referral | Handing over 42 Level I specialists to work in 26 disadvantaged and border districts. |

# **Supplementary Table 2. Included Studies and Extracted Data**

| **Country** | **Title** | **Study design** | **Description** | **Reported results** | **Reference** |
| --- | --- | --- | --- | --- | --- |
| Thailand | Mandatory rural service for health care workers in Thailand | Policy report | Reports strategies and their outcomes in supporting rural health care and the mandatory rural service | The conventional mandatory rural service began since Thailand's first medical school (in 1900), which evolved with new initiatives to improve its efficiency: penalty up to $10,000 for contract breach, mandating 3 years of rural public service for public medical school education fee (not applicable to private students) - which increased the doctor-to-population ratios in rural area from 1:7000 (1965) to 1:5850 (2010), prerequisite for specialist and postgraduation training, and monthly top of ($400) for rural physicians. The CPIRD, started in 1985 as an expansion of the MESRAP, recruited students from rural and remote areas. It recruited 1700 graduates per year in 2010, and almost all went to rural locations after graduation, with the retention rate after mandatory service reported to be two-thirds. Other incentives included scholarship by the Royal Thai Government to cover the total specialty training fee (approximately US$12,000), and eligibility for exams for board certifications that are recognized as equivalent to a degree at PhD level (applicable to physicians working over 5 years in rural areas). A monthly 'top up' amount ($400) for rural physicians who practice only in governmental units was also implemented, where adding to the baseline salary (approximately US$350), but still not as much as can be earned in a private hospital. | Wiwanitkit V. Mandatory rural service for health care workers in Thailand. RRH [Internet]. 2011 Feb 24; Available from: https://www.rrh.org.au/journal/article/1583 |
| Thailand | Equity of health workforce distribution in Thailand: an implication of concentration index | Cross sectional quantitative research | Assesses the equity of health workforce distribution in public hospitals through the application of “concentration index” (showing the contribution of rural policies) and discusses the successes of different iniatives | Between the employment years 2001 to 2015, a total of 19 338 medical graduates commenced work in the MOPH health services. Rural retention policies over the past four decades contributed to fair equitable distribution of HRH in Thailand across provincial economic status, as a slight pro-poor effect was found at district hospitals. Different initiatives had their roles and effectiveness in different contexts. Before the rapid economy growth, the conventional compulsory rural service policy was most successful in addressing external migration and rural shortage. When the econmic boom, additional strategies like rural recruitment, rural training, and hometown placement helped reverse the draining of physicians to private sectors. Financial incentives, despite some contributions, are not the only reason for retention as private hospitals almost always offer far higher salary. The prerequisite public service for specialist training appears to play significant role in attracting medical graduates to rural hospitals in recent years. The study also delineated a specific time of implementation of the compulsory rural service policy - 1972. Other special allowances for physicians were remote hardship, non-private practice, non-official hour service, and long years of service; combined could make the total monthly income 5–10 times of doctor basic salaries. | Witthayapipopsakul W, Cetthakrikul N, Suphanchaimat R, Noree T, Sawaengdee K. Equity of health workforce distribution in Thailand: an implication of concentration index. RMHP. 2019 Feb;Volume 12:13–22. |
| Thailand | Impact of a 20-year collaborative approach to increasing the production of rural doctors in Thailand | Commentary, perspective | Describes the establishment, strategies used and the impact of the ODOD and CPIRD on production of doctors in rural areas | Compared conventional versus special track of recruitment, concluded that a collaborative approach is effective and efficient.. The normal track (operated by the MOE) demanded 3-year compulsory service, with 11,300 USD fine imposed for non-compliance. The special track including ‘CPIRD’ and ‘ODOD’, established in 1994 and 2005 respectively, is a collaboration between the MOPH and MOE. The CPIRD are recruit students from rural domiciles, although they are mainly from secondary schools in the provincial areas, demanded 3-year mandatory service in MOPH hospitals, with penalty of 11,300 USD for non-adherence. The ODOD program extend to those from more targeted rural and remote areas, with full government scholarship to pursue 6-year medical training, have pre-specified job placement in their home districts and are liable for a 12-year long mandatory service in the MOPH service hospitals, with fine of 56,000 USD for non-adherence. In terms of result, up to 2015, the CPIRD/ODOD project has produced a total of 5,926 doctors for the country. 92% of graduates remained working in the provinces to which they were primarily assigned, and they accounted for 39.0% of all doctors entering community hospitals each year. Compared to normal track graduates, CPIRD/ODOD graduates appeared to have better clinical competencies and were twice as likely to fulfil their 3-year mandatory service and continue to work in the rural areas for a longer period after mandatory service. | Nithiapinyasakul A, Arora R, Chamnan P. Impact of a 20-year collaborative approach to increasing the production of rural doctors in Thailand. Int J Med Educ. 2016 Dec 22;7:414–6. |
| Thailand | Retention of doctors in rural health services in Thailand: impact of a national collaborative approach | Retrospective study | Investigates retention of medical graduates under the normal-track and special rural recruitment track commencing work in the MOPH hospitals between 2001 and 2015 and remained working up to 1 June 2016. Finds factors associated with retention. | The overall and year-by-year retention of medical graduates under the special recruitment track was higher than the normal track (overall retention of 78.2% and 52.5% respectively, p<0.05). Compared to their normal track counterparts, medical graduates under the special recruitment scheme were about 2.4-fold more likely to remain working in the MOPH health services for a minimum period of 3 years (OR 2.44, 95% CI 2.19-2.72). Among 4,869 medical graduates under the special recruitment track who remained working for the MOPH, 4425 (90.9%) still worked in the provinces to which they were primarily assigned. The ODOD program extends student recruitment to those from more targeted rural and remote areas, with full government scholarship given to pursue 6-year medical training.  The normal track graduates' workplace is based on individuals' choice provided each year's vacancy availability in governmental hospital (both in and outside of MOPH). The special track schools partnered with 37 accredited MOPH hospitals nationwide for clinical training, while normal track students took them in hospital universities. CPIRD recruits from mainly in mixed rural-urban provinces. | Arora R, Chamnan P, Nitiapinyasakul A, Lertsukprasert S. Retention of doctors in rural health services in Thailand: impact of a national collaborative approach. Rural Remote Health [Internet]. 2017 Sep 30;17(3). Available from: http://www.rrh.org.au/articles/subviewnew.asp?ArticleID=4344 |
| Thailand | Integrated strategies to tackle the inequitable distribution of doctors in Thailand: four decades of experience | Empirical research study | Summarizes strategies to solve inequitable distribution of human resources for health (HRH) between urban and rural areas, by using four decades of experience in Thailand as a case study for analysis | The first batch of compulsory-contracted Thai doctors started their assignments in 1972, which first started for physicians in 1967 in response to external brain drain. In the first four years (1967–1970) it was voluntary: the medical students chose either to work for 3 years in public facilities or pay a high annual tuition fee (USD400 per year for four years, or USD3000 at 1997 prices). If they breached the contract, they would have to pay a fine of USD4800. This fine was increased to USD8000 and USD16 000 in 1971 and 1973, respectively. Due to high social concern, high tuition fees and little opportunity for private practice, most medical students signed the contract. In 1972 this contract became compulsory for all medical students. Under CPIRD, students spend the first three preclinical years at medical schools (both central and regional) and the second three clinical years at 12 regional hospitals with the networking of district hospitals. Experience in rural public services is now a prerequisite for the residency training programmes. For physicians working over 5 years in rural areas, if they pass the examination, they receive a Certificate of Proficiency (called the Thai Board certificate) from the Medical Council of Thailand. This is offered in the specialty of preventive medicine, general practice and family medicine. The Civil Service Commission considers these Thai Board certificates equivalent to a Doctor of Public Health or Doctor of Philosophy degree. | Wibulpolprasert S, Pengpaibon P. Integrated strategies to tackle the inequitable distribution of doctors in Thailand: four decades of experience. Hum Resour Health. 2003 Nov 25;1(1):12. |
| Thailand | Rural physician recruitment program in Thailand: factors associated with medical student enrollment and post-graduation intentions | Cross-sectional survey | Studied the main reasons and the intention to work in rural areas of 2,870 4th–6th year medical students from 34 teaching hospitals under the CPIRD via questionnaire survey | The top three reasons for choosing to attend rural medical schools were: intention to return to work in their hometown (32%); student recruitment being less competitive than that of traditional medical schools (18.5%); and pre-specified job placement in their home districts (15%). The majority of medical students had a favorable attitude towards rural work, as defined by intention to work in rural health services for at least three years. 492 medical students (37%) reported that they intended to work in rural areas lifelong, and two-fifths intended to work in rural areas for three years as indicated in contract agreement. | Hongkan W, Muenpa R, Chamnan P. Rural physician recruitment program in Thailand: factors associated with medical student enrollment and post-graduation intentions. EfH. 2024 Jul 30;37(2):101–9. |
| Malaysia | Human Resources for Health Country Profile 2019-2021 Malaysia | Policy report | Provides information reflecting the human resources for health situation mainly from 2019 to 2021 in Malaysia, aims to improve future policy development and planning, and recommends measures involving HRH supply and demand in the country | Malaysia’ s regulatory framework requires medical graduates to complete a two-year housemanship within the public sector, followed by an additional two years of compulsory service. It notes that the extension of the housemanship period from one to two years in 2008 significantly reshaped the training landscape. Additionally, the introduction of a contract appointment system in 2016 stemmed from the limited number of available posts within the Ministry of Health (MOH), which had been further constrained by the government’ s broader human resource optimization policies. Specifically, since 2015, the implementation of the Policy on Optimisation of Human Resources in the Public Service and the Policy on Rationalisation of the Size of the Public Service restricted the creation of new permanent positions unless offset by existing vacancies.  It also highlights that administrative and other issues caused over 30% of house officers to exceed the stipulated two-year housemanship period, further delaying the release of permanent UD41 positions. This, coupled with the growing number of medical graduates, led to prolonged waiting times for housemanship placements. In response, the government introduced the contract appointment system in December 2016 for medical, dental, and pharmacy graduates. As described in the study, this mechanism was intended to shorten waiting times by allowing appointments independent of permanent post availability, while granting MOH flexibility to allocate training slots based on institutional capacity. Despite the shift to contract-based appointments, the study notes that opportunities remain for contract officers who have completed housemanship or compulsory service to secure permanent positions, depending on set criteria and vacancy availability. Overall, the study observes that the 2016 contract system helped reduce delays in housemanship and compulsory service placement for new graduates. | Planning Division, Ministry of Health Malaysia. Human Resources for Health Country Profile 2019-2021 Malaysia [Internet]. Level 6, Block E6, Parcel E Federal Government Administrative Centre 62590, Federal Territory of Putrajaya; 2023. Available from: https://www.moh.gov.my/moh/resources/Penerbitan/HRH/HRH_COUNTRY_PROFILES_2019-2021_FINAL.pdf |
| Malaysia | Contract doctors’ strike in Malaysia: A content analysis of the perception of medical fraternity and stakeholders on Facebook | Qualitative content analysis | Explores the perception of the stakeholders towards the doctor strike in Malaysia, to understand the underlying issues of contract appointment policy | Under the Malaysia’s five-year contract system, medical graduates fulfill their mandatory service through three years as house officers and two years as medical officers. It notes that between December 2016 and May 2021, only 3.4% of 23,077 contract medical officers received permanent appointments in the Ministry of Health (MOH) after completing their tenure. Previously, medical graduates were automatically absorbed into the civil service as permanent medical officers at grade UD44 upon completing housemanship, but under the contract system, they remain at grade UD41 — the lowest salary level for doctors — resulting in an annual pay gap of about RM8,000 compared to their UD44 counterparts. The study also highlights that contract officers are excluded from government scholarships for specialization, unrecorded leave, and government loans. Although pay adjustments in 2020 elevated their grade from UD41 to UD43, the study emphasizes that salary stagnation persists, as contract doctors face capped pay scales without time-based increments, leaving many struggling to support their families. | Jinah N, Lee KY, Zakaria NH, Zakaria N, Ismail M, Mohmad S. Contract doctors’ strike in Malaysia: A content analysis of the perception of medical fraternity and stakeholders on Facebook. Ooi PB, editor. PLOS ONE. 2023 Sept 28;18(9):e0292213. |
| Malaysia | Extended Executive Summary: Supply and Needs-Based Requirement Projections of Malaysian Human Resources for Health Using System Dynamics Approach (Doctor, Dentist, Pharmacist, Nurse, Assistant Medical Officer) 2016–2030 | Policy report | Simulates multiple scenarios to estimate future Malaysia’s HRH needs and supply under different policy and system parameters. | The study indicates that in Malaysia, medical graduates are required to complete a minimum of two years of clinical training—referred to as housemanship—before obtaining full registration with the Malaysian Medical Council (MMC). This period is designed to ensure their competency as medical practitioners. While various sources describe the duration of housemanship in Malaysia as ranging from one to three years, depending on policy changes and administrative factors, this particular report specifies a minimum two-year requirement, reflecting the standardized duration adopted in recent years. | Planning Division, Ministry of Health Malaysia. Extended Executive Summary: Supply and Needs-Based Requirement Projections of Malaysian Human Resources for Health Using System Dynamics Approach (Doctor, Dentist, Pharmacist, Nurse, Assistant Medical Officer) 2016–2030 [Internet]. Ministry of Health Malaysia; 2019. Report No.: MOH/S/RAN/149.19(AR)-e. Available from: https://www.moh.gov.my/moh/resources/Penerbitan/Laporan/Umum/Supply%20And%20Needs/mobile/index.html#p=2 |
| Malaysia | Permanent medical officers must choose a placement in Sabah or Sarawak | Official government news report / policy announcement (media release) | The news report outlines the Ministry of Health’ s introduction of a placement policy, which requires contract medical officers appointed to permanent positions to include at least one placement choice in rural area | The Ministry of Health (MOH) Malaysia introduced a new placement policy requiring contract medical officers who are appointed to permanent positions to select at least one of their three placement choices in Sabah or Sarawak (rural area) via the e-Placement 2.0 system. According to the report, this policy aims to address the uneven distribution of healthcare personnel between Peninsular Malaysia and the Borneo regions. It further notes that this measure represents the first initiative of its kind and reflects the government’s commitment to reducing disparities in the deployment of specialists, doctors, and nurses nationwide. Under this policy, the permanent placement quotas were set at 650 positions in Sarawak and 310 in Sabah, accounting for 42.7% of the 2,248 medical officers newly assigned across the country. | Berita RTM - Official Malaysian government news portal [Internet]. 2025. Permanent medical officers must choose a placement in Sabah or Sarawak – Ministry of Health. Available from: https://berita.rtm.gov.my/nasional/senarai-berita-nasional/senarai-artikel/pegawai-perubatan-tetap-wajib-pilih-penempatan-di-sabah-atau-sarawak? |
| Malaysia | Health Workforce Crisis: Recruitment and Retention of Skilled Heath Workers in the Public Health Sector in Malaysia | Policy review | Discusses worldwide policy initiatives that respond to the health care workforce shortage and health service delivery in different countries. Five policy initiatives are discussed and related to the Malaysian context | In most remote states (Sabah and Sarawak) - Regional Incentive Payment of additional 12.5 - 25% of monthly salary, while other rural areas receive Rural Incentive Payment of additional 10% of salary. Additional incentives in housing, car loans. Allowances:duty allowance, relocation allowance, allowance while attend professional development program. For medical practitioners, the three pathways available are clinical specialist training in various disciplines under a Federal government scholarship, medical administration and non-specialist clinical medical officers. There are also opportunities to attend courses and further training overseas (MOH 2006). Pre requisites to gaining entry into medical specialty training are confirmation of service in the public sector and satisfactory work performance. | Abdul Rahim R, Mwanri L. Health Workforce Crisis: Recruitment and Retention of Skilled Health Workers in the Public Health Sector in Malaysia. Asia Pacific Journal of Public Administration. 2012 Dec;34(2):157–70. |
| Malaysia | Malaysia Health System Review | Policy report | A country-based report that provides a detailed description of a health system and of reform and policy initiatives in progress or under development in Malaysia | Malaysia’ s public service career pathway for doctors before the contract doctor system: after completing the two-year housemanship, medical officers are automatically promoted from grade UD41 to UD44 within the civil service grading system. Those who subsequently complete their two-year compulsory public service are then advanced to grade UD48. Additionally, doctors who pass their postgraduate specialist examinations are promoted to UD48 and may progress further to UD52 upon receiving official recognition as specialists. | Safurah Jaafar, Kamaliah Mohd Noh, Khairiyah Abdul Muttalib, Nour Hanah Othman, Judith Healy, Kalsom Maskon, et al. Malaysia Health System Review [Internet]. Asia Pacific Observatory on Health Systems and Policies, World Health Organization, Regional Office for the Western Pacific; 2013. (Health Systems in Transition). Report No.: Vol.3 No. 1. Available from: https://iris.who.int/server/api/core/bitstreams/8f9ae21a-a591-44a9-944d-5ab02370ecb4/content |
| Malaysia | House officers’ specialist career choices and motivators for their choice– a sequential mixed-methods study from Malaysia | Sequential mixed-methods study | Identify the house officers’ medical specialty career choices and qualitatively explore the motivational factors related to the domains of personal, professional and career prospects. | The recent establishment of numerous private medical colleges in Malaysia has led to an oversupply of medical graduates, resulting in approximately one thousand graduates waiting nearly a year for job placements. It further reports that only about 45–50% of those completing housemanship are offered permanent positions, with the majority of medical officers appointed on a contractual basis and ineligible for government-sponsored postgraduate training support. According to the study, these conditions may significantly influence the career choices and long-term decisions of medical graduates. | Nadarajah A, Shankar PR, Jayaraman S, Sreeramareddy CT. House officers’ specialist career choices and motivators for their choice– a sequential mixed-methods study from Malaysia. BMC Med Educ. 2022 Nov 16;22(1):796. |
| Malaysia | Alternate careers for medical graduates and house officers in Malaysia. | Descriptive quantitative study | Explore perceptions, awareness, and interest in alternative career options of medical graduates in Malaysia | Malaysia produces an average of 5,000 medical graduates annually. However, only around 4,000 graduates are absorbed each year, leaving a portion of graduates unemployed for up to eight months or longer while awaiting job placements. Among those entering the housemanship programme, the study notes that only 40–50% are eventually offered permanent civil service positions. In recent cohorts, nearly all housemen completing training have been appointed as contract medical officers (MOs) at UD41 grade, contrasting with earlier years when most graduates were promptly absorbed and granted permanent positions upon completing housemanship. The government expanded the number of training hospitals from 38 to 47 and increased training positions from 10,835 to 11,706 across the country. Additionally, by shifting from permanent to contract appointments, the Ministry of Health aimed to allow trainees to complete housemanship and obtain full registration under the Medical Act 1971, while expecting that some would later transition to private practice—thereby freeing up vacancies for new house officers. | Naicker AS, Vinoshn DK, Yuliawiratman BS, Alaga A, Kevin Ng WS, Naicker MS, et al. Alternate careers for medical graduates and house officers in Malaysia. Med J Malaysia. 2021 Mar;76(2):183–9. |
| Malaysia | Annual Report 2016, Ministry of Health, Malaysia | Policy report | Presents the Ministry of Health Malaysia’s key activities, policies, and performance indicators for 2016, covering public health programmes, healthcare service delivery, human resources for health, infrastructure development, and health financing | The report explains that the Ministry of Health (MOH) Malaysia continued to face a shortage of specialists, particularly in Sabah and Sarawak, and therefore maintained efforts to reappoint retired specialists to fill critical service gaps. It notes that while the MOH decided not to employ foreign medical officers, Malaysian citizens could still be appointed on a contractual basis to complete their compulsory service, depending on post availability. The report also highlights that contract renewals were extended to medical officers who had obtained postgraduate specialist qualifications during the gazettement process, as well as to house officers completing their housemanship training. Oversight of these appointments was managed by the MOH. According to the report, in 2016, a total of 144 doctors were employed on a contractual basis, compared to 268 in 2015, reflecting the ministry’s continued reliance on contract appointments to address workforce shortages. | Ministry of Health Malaysia. Annual Report 2016 [Internet]. 2016. Report No.: MOH/S/RAN/119.18(R). Available from: https://govdocs.sinarproject.org/documents/ministry-of-health/annual-reports/annual-report-2016.pdf/view |
| Malaysia | Malaysian Medical Students’ Career Intention (MMSCI): a cross-sectional study | Descriptive cross-sectional study | Determines the current Malaysian medical students’ career intentions immediately after graduation and upon completing the 2 years of housemanship and to establish the factors influencing these intentions. | Specialist training opportunities in Malaysia have become increasingly uncertain in recent years due to the contractual appointment system for medical officers, which initially did not include eligibility for government-sponsored specialist training prior to 2022. It further explains that although limited training seats were made available to contract medical officers starting in 2022, the restricted access has prompted many medical graduates to explore alternative pathways for career advancement. | Teng ZS, Ser GTZ, Hong WH, Teo CH, Abdul Aziz YF, Vadivelu J, et al. Malaysian Medical Students’ Career Intention (MMSCI): a cross-sectional study. Hum Resour Health. 2024 Aug 22;22(1):59. |
| Indonesia | What do doctors want? developing incentives for doctors to serve in Indonesia's rural and remote areas | Policy Research Working Paper | Analyze the locations chosen by graduating medical students before and after a major change in the incentive system, and survey data on choices among hypothetical assignments differing in compensation, career prospects, and amenities at various locations suggest that the current policy of offering specialist training is incentive enough to make doctors from Java willing to serve in remote areas | Location-based bonuses and career advancement opportunity in civil service recruitment. | Chomitz K, Setiadi G, Azwar A, Ismail N, idiyarti. What Do Doctors Want? Developing Incentives for Doctors to Serve in Indonesia’s Rural and Remote Areas [Internet]. The World Bank; 1999. (Policy Research Working Papers). Available from: http://elibrary.worldbank.org/doi/book/10.1596/1813-9450-1888 |
| Indonesia | New Insights into the Provision of Health Services in Indonesia: A Health Workforce Study | Policy report | Describes the current health status, policies and their impact on the supply and distribution of the health workforce. The report also provide policy suggestions | The PTT (Pegawai Tidak Tetap, or contract employee) program was introduced in 1992. Under this program, newly graduated doctors were no longer immediately hired as civil servants but were required first to fulfill a nonrenewable, three-year appointment as “nonpermanent employees” in a province where a post became available. The PTT program was formally abolished in 2007.Via the program, each year more than 1,000 doctors spread to remote and very remote areas from the 1992 to 2002. To 2007, 1,500 doctors were serving in remote and very remote areas. Those PTT doctors who had served in remote or very remote areas through the PTT scheme would generally receive priority in the subsequent civil service recruitment process. The public sector only absorbed about 40 percent through the PTT program. In 2007, a new policy was established offering a 6-month period of service in remote areas for new graduates and an attractive salary package. In addition to the base salary, the new graduates received a monthly bonus amounting to as much as two-and-a-half times the base for very remote postings. This incentive package (generous remuneration and a short term of service), combined with a growing number of medical graduates entering the labor market after an expansion in the number of medical schools, has increased the interest of graduates in rural and remote postings. In addition, service as a PTT contract doctor in a remote area still contributes to positive evaluation for recruitment into the civil service. Deployment (Table 3.2)~1,000 doctors/ year (1992 - 2006) and 1,595 doctors/ year (2006 - 2007) via PTT program. | Rokx C, Giles J, Satriawan E, Marzoeki P, Harimurti P. New Insights into the Supply and Quality of Health Services in Indonesia [Internet]. The World Bank; 2010. Available from: https://hdl.handle.net/10986/2434 |
| Indonesia | Health worker recruitment and deployment in remote areas of Indonesia | Policy report | Elaborated on the policy efforts to address the shortage and uneven distribution of health workers in Indonesia’s remote areas | Newly graduated doctors after PTT compulsory service, choose between (1) further specialize, (2) become a civil servant (exam required), (3) enter private sector. Results: 1992 to 2002, each year more than 1000 doctors spreads to remote and very remote area via PTT program. To 2007: 1500 doctors serve in remote and very remote areas. (Because of bonus in salary). Table 2: monthly incentives and salary (A medical specialist who serves in a very remote area earns 10,350,000 IDR or approximately 1150 USD per month (~1 USD = 9000 IDR). Table 3: Number of staff per year. Moreover, in 2010, only 17% of the 9000 very remote health centers were without a doctor, compared with 30% of 8000 health centers in 2006. | Efendi F. Health worker recruitment and deployment in remote areas of Indonesia. RRH [Internet]. 2012 Jun 5; Available from: https://www.rrh.org.au/journal/article/2008 |
| Philippines | Factors affecting retention in the Philippine National Rural Physician Deployment Program from 2012 to 2019: a mixed methods study | Descriptive, mix-methods, explanatory design | Identify individual, work-related, local, national, and international factors influencing the Philippine physicians in the Barrios program from 2012 to 2019 through surveys and interviews, and propose interventions for improving retention in rural areas | DTTBs was established in 1993, with a duration of 2 years. Incentives include competitive pay (gross monthly compensation USD 1870), additional hazard pay (if areas with humanitarian conflict); subsistence & laundry, housing & insurance (other benefits as described in Magna Carta of Public Health Workers); career advancement: Scholarship for Master's degree in Public Management. After 2 years, can practice as epidemiologist, do public health research, or undergo residency training (public or private hospital), or remain in assigned municipality. | Flores ELL, Manahan EMR, Lacanilao MPB, Ladaw IMaBT, Mallillin MMB, Mappatao NTQ, et al. Factors affecting retention in the Philippine National Rural Physician Deployment Program from 2012 to 2019: a mixed methods study. BMC Health Serv Res. 2021 Dec;21(1):1201. |
| Philippines | Cost-effectiveness analysis of a physician deployment program to improve access to healthcare in rural and underserved areas in the Philippines | Cost-effectiveness analysis (CEA) | Detailed analysis of the cost-effectiveness (in terms of lives saved and quality-adjusted life years (QALYs) gained) and incremental cost-effectiveness ratio (ICER) of physician rural deployment programs in reducing pediatric pneumonia and diarrhea mortality | Incentives include higher-than-average salary, living and transportation allowances, hazard pay, professional development trainings. Integration as it recruits medical doctors (primarily general practitioners, though other primary care physicians also deployed). Purpose: 17.5x higher physician density in urban compareed to rural area as in the 2019 Universal Health Care Act, propose a National Health Workforce Support System, scale up HRH deployment program like DTTB. | Avanceña ALV, Tejano KPS, Hutton DW. Cost-effectiveness analysis of a physician deployment program to improve access to healthcare in rural and underserved areas in the Philippines. BMJ Open. 2019 Dec;9(12):e033455 |
| Philippines | Assessment of factors influencing retention in the Philippine National Rural Physician Deployment Program | Mix-methods (questionnaires + oral interview) | Reports the factors impacting decisions of deployed physician to remain in rural sites, suggesting the need for improvement in policy formation | Vision of the program is that all municipalities in Philippine would have a doctor within 20 years, deployed DTTBs to 271 municipalities without doctors. After 2 years, they have a choice to remain or Physician of DTTB assume the role of Municipal Health Officer (MHO) (but have a drop in total compensation upon this absorption. Between 1993 - 2011: 452 DTTBs took part, only 81 (18%) chose to remain. | Leonardia JA, Prytherch H, Ronquillo K, Nodora RG, Ruppel A. Assessment of factors influencing retention in the Philippine National Rural Physician Deployment Program. BMC Health Serv Res. 2012 Dec;12(1):411. |
| Philippines | Impact Evaluation of the Human Resource for Health Deployment Program (HRHDP) | Policy Analysis | Describes the impact of DTTB deployment on health indicators, such as contraceptive use, nutrition, life expectancy at birth, and healthcare access in rural areas | Impact on health indicators: DTTB deployment doubles the odds of using modern contraceptives among poor women and decreases the odds of being underweight by two-thirds among poor children; life expectancy at birth among averted underweight children. In DTTB-recipient municipalities is projected to increase by 3.5 years. The overall estimated internal rate of return of 15.9%, which is likely to be severely understated. In terms of health access, increases the probability of having a public health center physician in the municipality by around 33 percentage points. Physician received a monthly salary of PhP88,410 (in 2022; salary grade 24), excluding additional benefits, which is significantly above the median salary for similarly experienced physicians. | Abrigo MRM, Opiniano GA, Tam ZC. Impact evaluation of the Human Resource for Health Deployment Program (HRHDP) [Internet]. Philippines: Philippine Institute for Development Studies; February 2023. Available from: https://hdl.handle.net/10419/284602 |
| Vietnam | Decision No. 585/QĐ-BYT of 2013 approving the Project "Pilot Deployment of Young Volunteer Doctors to Work in Mountainous, Remote, Border, Island Areas, and Regions with Especially Difficult Socio-Economic Conditions (Priority Given to 62 Poor Districts)" issued by the Minister of Health. | Legal document | Decision of the Vietnamese Ministry of Health to issue Decree 585 in 2013 | Decision/ Project 585 in Vietnam aims to recruit and train doctors to work in rural and underserved areas. The duration of the program is 3 years for male doctors and 2 years for female doctors. The incentive structures include the same salary and benefits as other staff in the institutions, along with financial support in the form of allowances. Additionally, the program provides an opportunity for specialist training, specifically for obtaining a Specialist Level I qualification, and guarantees a position in the hospital. The implementation of the project involves freshly graduated MDs and specialist doctors, with two main phases: Phase 1, which took place from 2013 to 2016, involved about 500 young volunteer doctors, while Phase 2, from 2016 onwards, focused on developing legal documents regarding the responsibilities and social obligations of young medical professionals. In terms of outcomes, Phase 1 (2015-2020) successfully trained 354 doctors, and Phase 2 (2021-2030) is set to train 278 doctors. | Ministry of Health of Vietnam. Decision No. 585/QD-BYT on the pilot program of deploying young doctors to work in remote, mountainous, border, and island areas with difficulties. Ministry of Health of Vietnam; 2013 |
| Vietnam | Decision No. 4359/QĐ-BYT of 2020 amending Decision No. 585/QĐ-BYT on the approval of the Project "Pilot Deployment of Young Volunteer Doctors to Work in Mountainous, Remote, Border, Island Areas, and Regions with Especially Difficult Socio-Economic Conditions (Priority Given to 62 Poor Districts)" issued by the Minister of Health. | Legal document | Decision of the Vietnamese Ministry of Health to issue Decree 4919 in 2020, an supplement to decree 585 on 2013, and their extended policies | Decision 4919 supplements the contents of Decision 585 with a clear purpose and vision aimed at strengthening rural healthcare. The decree sets a target to deploy 450 young doctors by 2025 and approximately 600 young doctors by 2030. It also stipulates that specialized doctor (Level I) must serve a minimum of 5 years. The purpose of the decree is to provide a supportive environment for new doctors to apply their knowledge in practice, ease the burden on higher-level healthcare facilities, and support lower-level facilities in providing healthcare services. Additionally, the decree aims to implement policies such as universal health insurance and rural development to improve overall healthcare accessibility and quality. | Ministry of Health of Vietnam. Decision No. 4359/QD-BYT amending Decision No. 585/QD-BYT on the deployment of young doctors to work in remote, mountainous, border, and island areas with difficulties [Internet]. Ministry of Health of Vietnam; 2020. Available from: https://thuvienphapluat.vn/van-ban/The-thao-Y-te/Quyet-dinh-4359-QD-BYT-2020-sua-doi-Quyet-dinh-585-QD-BYT-dua-Bac-sy-tre-cong-tac-tai-mien-nui-456023.aspx |
| Vietnam | Handing over 42 Level I specialists to work in 26 disadvantaged and border districts. | Evaluation of policy implementation | Report on the total number of physicians deployed from 2013-2024 under Project 585 | To date, Project 585 has received and trained a total of 699 Level I specialist doctors who are working in 155 disadvantaged, border, and island districts across 39 provinces. After training, the project has handed over 444 doctors to 89 disadvantaged, border districts in 22 provinces in the northern mountainous region, central region, and Central Highlands. | Ministry of Health of Vietnam. Handing over 42 Level I specialists to work in 26 disadvantaged and border districts [Internet]. Ministry of Health of Vietnam; 2024. Available from: https://moh.gov.vn/hoat-dong-cua-dia-phuong/-/asset_publisher/gHbla8vOQDuS/content/ban-giao-42-bac-si-chuyen-khoa-cap-i-ve-cong-tac-tai-26-huyen-kho-khan-bien-gioi |

# **Supplementary Table 3. Result Summary**

**Comparison of Rural Physician Deployment Program in Five Southeast Asian countries**

| **Country** | | **Thailand** | | | **Malaysia** | **Indonesia** | **Philippines** | **Vietnam** |
| --- | --- | --- | --- | --- | --- | --- | --- | --- |
| **Name of Program(s)** | | Conventional Track: Compulsory Rural Service | Special Track: Collaborative Project to Increase Production of Rural Doctors (CPIRD) | Special Track: One District One Doctor (ODOD) | Compulsory Public Service Program | Temporary Employment Program (Pegawai Tidak Tetap – PTT & Nusantara Sehat – NS) | Doctors-to-the-Barrios (DTTB) | Decision 585/2013 |
| **Year of Implementation** | | 1972^1^ | 1994^2^ | 2005^2^ | 1971^3^ (Medical Act)  2016^3–5^ (Contract Doctor System) | 1992^6^ | 1993^7^ | 2013^8^ |
| **Is Participation Mandatory?** | | Yes  (for students in public universities)^2^ | No | No | Yes | No  (since 2007)^6^ | No | No |
| **Duration** | | 3 years^2^ | 3 years | 12 years | 2 years^9,10^ | 3 years (before 2007)  6 months (since 2007)^6^ | 2 years^7^ | 5 years^8,11^ |
| **Incentives** | **Career Promotion** | Eligible for residency after 3 years; eligible for PhD-equivalent exam (Certificate of Proficiency) after 5 years^2,12^ | | | Automatic promotion to UD-48 (a career grade) before 2015^13^; post-2016 contract officers remain at UD41–43 without time-based increments^9^ | Given priority in subsequent civil service recruitment^6,14^ | After 2 years, eligible to practice as epidemiologist, conduct public health research, pursue residency (public/private hospital), or remain in assigned municipality^7^ | Eligible for 20-month postgraduate training; guaranteed local hospital position upon completion; priority in postgraduate admission^8^ |
|  | **Scholarship** | Eligible for Royal Thai Government scholarship covering full specialty training fee (~US$12,000) and PhD-equivalent degree in preventive medicine, general practice, and family medicine after 5 years of rural service^2,12^ | | | Eligible for federal scholarships for specialist training^13^; but limited seats under the contract doctor system^9,15^ | Eligible for scholarships for specialist training^6^ | Eligible for scholarship for a Master’s degree in Public Management^7^ | Tuition covered for licensing and specialist training (Level I, II)^8^ |
|  |  |  | | Full scholarship (tuition & living) for medical training^16^ |  |  |  |  |
|  | **Salary** | Monthly top-up of $400 in addition to baseline salary (~$350); maximum up to $1,379/month (still lower than private practice)^2,12^ | | | $1,050/month^9^, additional 10–25% salary increase based on remoteness of posting^13^ | $600–$1,150/month, depending on remoteness and qualifications^17^ | $1,870/month (above national average)^7,18^ | Same salary and benefits as other staff in these institutions^8^ |
|  | **Allowance** | Remote hardship, non-private practice, after-hours service, and long-service allowances; combined can reach 5–10× basic salary^1^ | | | Duty allowance, relocation support, and housing loan^13^ | Duty allowance, relocation support, and housing loan^6,14,17^ | Subsistence, laundry, housing, insurance, and transportation benefits (per Magna Carta of Public Health Workers)^7,18^ | Travel reimbursements, performance bonuses, on-call payments, housing and living support, land allocation, Party membership^8^ |
| **Results** | **Average number of physicians deployed per year (doctors per year) (*)** | ~894.73 (**) | ~393.93 (***) | | (Data not found) | ~1,000 (before 2007)  1,595 (since 2007)^6^ | ~23.78 (****) | ~70 (*****) |
|  | **Retention rate** | 52.50%^16^ | 78.20%^16^ | | (Data not found) | (Data not found) | 18%^19^ | (Data not found) |
| **Impacts on rural health** | **Healthcare accessibility & coverage** | Increased doctor-to-population ratio in rural areas from 1:7,000 (1965) to 1:5,850 (2010)^2^ | Special-track graduates are 2.4× more likely to remain at MOPH for ≥3 years compared to normal track^16,20^ | | (Data not found) | Improved availability of health workers in health centers and villages, especially in remote areas; in 2010, only 17% of 9,000 very remote centers lacked a doctor vs. 30% of 8,000 centers in 2006^17^ | Increased likelihood of having a public health center physician in the municipality by ~33 percentage points^21^ | Deployed 444 doctors to 89 disadvantaged, border districts in 22 provinces (northern mountainous, central, and Central Highlands regions)^22^ |
|  | **Improvements in rural health indicators** | (Direct impacts of these program in this regard are not found) | | | (Direct impacts of these program in this regard are not found) | (Direct impacts of these program in this regard are not found) | 2× contraceptive use in poor women; ⅔ lower underweight risk in poor children^21^ | (Direct impacts of these program in this regard are not found) |

*(*) The average number of physicians deployed by each program is calculated via the following formula:*

$$Average number of physicians deployed per year=\frac{Total number of physicians deployed}{Number of years}$$

*Except for that of PTT by Indonesia, in which the average numbers of physicians deployed each year to remote and very remote areas was directly derived from Table 3.2 of article (4) in the reference.*

*(**) Within a 15-year period (from 2001 to 2015), approximately 13,421 (69.4% of a total of 19 338) medical graduates commenced work in the MOPH health services via the normal rural recruitment track (1). Applying the formula (*), the average number of physicians deployed by both the CPIRD and ODOD of Thailand was calculated.*

*(***) Within a 15-year period (from 2001 to 2015), a total of 5909 medical graduates commenced work in the rural areas via the Ministry of Public Health programs, special rural recruitment track (1). Applying the formula (*), the average number of physicians deployed by both the CPIRD and ODOD of Thailand was calculated.*

*(****) Within a 19-year period (from 1993 to 2011), a total of 452 DTTBs took part in the program (2). Applying the formula (*), the average number of physicians deployed by DTTB of Philippines was calculated.*

*(*****) Within a 12-year period (from 2013 to 2024), a total of 699 doctors were enrolled via the Decree 585 (3). Applying the formula (*), the average number of physicians deployed by Decree 585 of Vietnam was calculated.*

# **References**

1.	Witthayapipopsakul W, Cetthakrikul N, Suphanchaimat R, Noree T, Sawaengdee K. Equity of health workforce distribution in Thailand: an implication of concentration index. *Risk Manag Healthc Policy*. 2019;Volume 12:13-22. doi:10.2147/RMHP.S181174

2.	Wiwanitkit V. Mandatory rural service for health care workers in Thailand. *Rural Remote Health*. Published online February 24, 2011. doi:10.22605/RRH1583

3.	Naicker AS, Vinoshn DK, Yuliawiratman BS, et al. Alternate careers for medical graduates and house officers in Malaysia. *Med J Malaysia*. 2021;76(2):183-189.

4.	Nadarajah A, Shankar PR, Jayaraman S, Sreeramareddy CT. House officers’ specialist career choices and motivators for their choice– a sequential mixed-methods study from Malaysia. *BMC Med Educ*. 2022;22(1):796. doi:10.1186/s12909-022-03845-2

5.	Ministry of Health Malaysia. *Annual Report 2016*.; 2016. https://govdocs.sinarproject.org/documents/ministry-of-health/annual-reports/annual-report-2016.pdf/view

6.	Rokx C, Giles J, Satriawan E, Marzoeki P, Harimurti P. *New Insights into the Supply and Quality of Health Services in Indonesia*. The World Bank; 2010. doi:10.1596/978-0-8213-8298-1

7.	Flores ELL, Manahan EMR, Lacanilao MPB, et al. Factors affecting retention in the Philippine National Rural Physician Deployment Program from 2012 to 2019: a mixed methods study. *BMC Health Serv Res*. 2021;21(1):1201. doi:10.1186/s12913-021-07219-0

8.	Ministry of Health of Vietnam. Decision No. 585/QD-BYT on the pilot program of deploying young doctors to work in remote, mountainous, border, and island areas with difficulties. Published online 2013.

9.	Jinah N, Lee KY, Zakaria NH, Zakaria N, Ismail M, Mohmad S. Contract doctors’ strike in Malaysia: A content analysis of the perception of medical fraternity and stakeholders on Facebook. Ooi PB, ed. *PLOS ONE*. 2023;18(9):e0292213. doi:10.1371/journal.pone.0292213

10.	Planning Division, Ministry of Health Malaysia. *Human Resources for Health Country Profile 2019-2021 Malaysia*.; 2023. https://www.moh.gov.my/moh/resources/Penerbitan/HRH/HRH_COUNTRY_PROFILES_2019-2021_FINAL.pdf

11.	Ministry of Health of Vietnam. Decision No. 4359/QD-BYT amending Decision No. 585/QD-BYT on the deployment of young doctors to work in remote, mountainous, border, and island areas with difficulties. Published online 2020. https://thuvienphapluat.vn/van-ban/The-thao-Y-te/Quyet-dinh-4359-QD-BYT-2020-sua-doi-Quyet-dinh-585-QD-BYT-dua-Bac-sy-tre-cong-tac-tai-mien-nui-456023.aspx

12.	Wibulpolprasert S, Pengpaibon P. Integrated strategies to tackle the inequitable distribution of doctors in Thailand: four decades of experience. *Hum Resour Health*. 2003;1(1):12. doi:10.1186/1478-4491-1-12

13.	Abdul Rahim R, Mwanri L. Health Workforce Crisis: Recruitment and Retention of Skilled Health Workers in the Public Health Sector in Malaysia. *Asia Pac J Public Adm*. 2012;34(2):157-170. doi:10.1080/23276665.2012.10779392

14.	Chomitz K, Setiadi G, Azwar A, Ismail N, idiyarti. *What Do Doctors Want? Developing Incentives for Doctors to Serve in Indonesia’s Rural and Remote Areas*. The World Bank; 1999. doi:10.1596/1813-9450-1888

15.	Teng ZS, Ser GTZ, Hong WH, et al. Malaysian Medical Students’ Career Intention (MMSCI): a cross-sectional study. *Hum Resour Health*. 2024;22(1):59. doi:10.1186/s12960-024-00939-4

16.	Arora R, Chamnan P, Nitiapinyasakul A, Lertsukprasert S. Retention of doctors in rural health services in Thailand: impact of a national collaborative approach. *Rural Remote Health*. 2017;17(3). doi:10.22605/RRH4344

17.	Efendi F. Health worker recruitment and deployment in remote areas of Indonesia. *Rural Remote Health*. Published online June 5, 2012. doi:10.22605/RRH2008

18.	Avanceña ALV, Tejano KPS, Hutton DW. Cost-effectiveness analysis of a physician deployment program to improve access to healthcare in rural and underserved areas in the Philippines. *BMJ Open*. 2019;9(12):e033455. doi:10.1136/bmjopen-2019-033455

19.	Leonardia JA, Prytherch H, Ronquillo K, Nodora RG, Ruppel A. Assessment of factors influencing retention in the Philippine National Rural Physician Deployment Program. *BMC Health Serv Res*. 2012;12(1):411. doi:10.1186/1472-6963-12-411

20.	Hongkan W, Muenpa R, Chamnan P. Rural physician recruitment program in Thailand: factors associated with medical student enrollment and post-graduation intentions. *Educ Health*. 2024;37(2):101-109. doi:10.62694/efh.2024.8

21.	Philippine Institute for Development Studies, Abrigo MR, Opiniano G, Tam Z, Department of Health. *Impact Evaluation of the Human Resource for Health Deployment Program (HRHDP)*. Philippine Institute for Development Studies; 2023. doi:10.62986/dp2023.03

22.	Ministry of Health of Vietnam. Handing over 42 Level I specialists to work in 26 disadvantaged and border districts. Published online 2024. https://moh.gov.vn/hoat-dong-cua-dia-phuong/-/asset_publisher/gHbla8vOQDuS/content/ban-giao-42-bac-si-chuyen-khoa-cap-i-ve-cong-tac-tai-26-huyen-kho-khan-bien-gioi
